# Pharmacokinetics of a single oral dose of vitamin D3 (70,000 IU) in pregnant and non-pregnant women

**DOI:** 10.1186/1475-2891-11-114

**Published:** 2012-12-27

**Authors:** Daniel E Roth, Abdullah Al Mahmud, Rubhana Raqib, Robert E Black, Abdullah H Baqui

**Affiliations:** 1 Department of International Health, The Johns Hopkins Bloomberg School of Public Health, Baltimore, MD 21205, USA; 2 International Center for Diarrhoeal Disease Research, Bangladesh (icddr,b), Dhaka, Bangladesh; 3 Current address: Department of Paediatrics, Hospital for Sick Children and University of Toronto, Toronto, ON, Canada

**Keywords:** Vitamin D, Bangladesh, Pregnancy, Pharmacokinetics

## Abstract

**Background:**

Improvements in antenatal vitamin D status may have maternal-infant health benefits. To inform the design of prenatal vitamin D3 trials, we conducted a pharmacokinetic study of single-dose vitamin D3 supplementation in women of reproductive age.

**Methods:**

A single oral vitamin D3 dose (70,000 IU) was administered to 34 non-pregnant and 27 pregnant women (27 to 30 weeks gestation) enrolled in Dhaka, Bangladesh (23°N). The primary pharmacokinetic outcome measure was the change in serum 25-hydroxyvitamin D concentration over time, estimated using model-independent pharmacokinetic parameters.

**Results:**

Baseline mean serum 25-hydroxyvitamin D concentration was 54 nmol/L (95% CI 47, 62) in non-pregnant participants and 39 nmol/L (95% CI 34, 45) in pregnant women. Mean peak rise in serum 25-hydroxyvitamin D concentration above baseline was similar in non-pregnant and pregnant women (28 nmol/L and 32 nmol/L, respectively). However, the rate of rise was slightly slower in pregnant women (i.e., lower 25-hydroxyvitamin D on day 2 and higher 25-hydroxyvitamin D on day 21 versus non-pregnant participants). Overall, average 25-hydroxyvitamin D concentration was 19 nmol/L above baseline during the first month. Supplementation did not induce hypercalcemia, and there were no supplement-related adverse events.

**Conclusions:**

The response to a single 70,000 IU dose of vitamin D3 was similar in pregnant and non-pregnant women in Dhaka and consistent with previous studies in non-pregnant adults. These preliminary data support the further investigation of antenatal vitamin D3 regimens involving doses of ≤70,000 IU in regions where maternal-infant vitamin D deficiency is common.

**Trial registration:**

ClinicalTrials.gov (NCT00938600)

## Background

Vitamin D is essential for the growth and development of the human skeleton throughout the life cycle [1]. There is considerable speculation regarding the potential effects of vitamin D on both skeletal and extra-skeletal aspects of reproductive physiology and fetal development, yet it remains unknown whether there are benefits to improving maternal antenatal vitamin D status beyond the correction of severe deficiency [2,3]. Clinical trials employing vitamin D dose regimens that safely optimize maternal-fetal vitamin D status will enable testing of these hypotheses [4]. However, very few studies have rigorously addressed vitamin D supplementation during pregnancy, and the single-dose vitamin D3 pregnancy trials published to date have provided little insight into pharmacokinetics or safety [5,6]. Moreover, there is a near complete absence of pharmacological data in South Asia, where the vitamin D status of pregnant women [7] and young infants [8] is poor in spite of the tropical climate.

The pharmacokinetics of oral vitamin D3 are conventionally described with respect to its effect on the serum concentration of the predominant circulating metabolite, 25-hydroxyvitamin D ([25(OH)D]), which is a well-established biomarker of systemic vitamin D status [9]. The present study was conducted to assess changes in serum [25(OH)D] and calcium following a single oral vitamin D3 dose (70,000 IU) in non-pregnant women and pregnant women in the third trimester of pregnancy in Dhaka, Bangladesh. The aim was to generate preliminary pharmacokinetic (PK) and safety data to inform the design of supplementation regimens for use in future larger-scale trials of antenatal vitamin D supplementation in Bangladesh.

## Methods

### Participants

**Figure 1:**
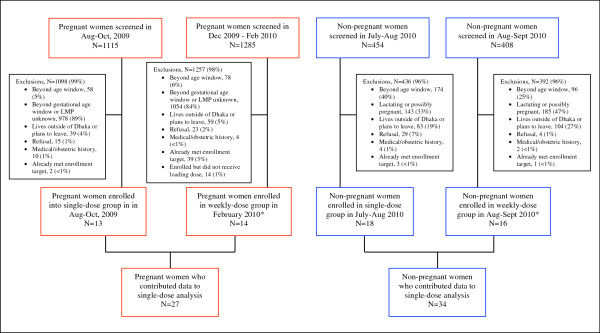
Flow diagram of participant screening, exclusions, and enrollment.

Pregnant and non-pregnant women were enrolled at a clinic in Dhaka, Bangladesh (24°N) from July 2009 to February 2010 if they were aged 18 to <35 years, held permanent residence in Dhaka at a fixed address, and planned to stay in Dhaka for at least four months (Figure 1). Reasons for exclusion were a known medical condition, self-reported current use of any dietary supplements containing vitamin D, use of anti-convulsant or anti-mycobacterial medications, severe anemia (hemoglobin concentration <70 g/L), or hypertension at enrollment (systolic blood pressure ≥140 mmHg or diastolic blood pressure ≥90 mmHg on at least two measurements). Pregnant women were excluded if they had major risk factors for preterm delivery (e.g., preterm labor or previous preterm delivery), pregnancy complications or had previously delivered an infant with a congenital anomaly or perinatal death. Non-pregnant women were excluded if they were possibly pregnant (e.g., missed recent menses) or lactating.

All participants in this study received a dose of vitamin D (70,000 IU) at baseline. Primary PK analyses involved participants who did not receive any additional vitamin D throughout follow-up (“single-dose group”). However, to enhance the assessment of 25(OH)D response and safety during the first week of follow-up, an additional cohort of participants who continued to receive weekly vitamin D doses beginning on day 7 (“weekly-dose group”) contributed biochemical data to the present analysis for the first 7 days after the 70,000 IU dose (i.e., up to the time *preceding* their 2nd dose). Findings related to the effect of weekly dosing will be reported elsewhere. Participants were enrolled in stages according to a design that enabled interim analyses and the testing of supplementation regimens in non-pregnant participants prior to their initiation in pregnant women: non-pregnant participants were enrolled in the summer (July to September 2009); pregnant women who received only a single dose were enrolled during the 30th week of gestation in August-September 2009; and, pregnant participants who received the initial dose followed by weekly doses were enrolled at 27 to <31 completed weeks of gestation in February 2010. The study was reviewed and approved by the Institutional Review Board at The Johns Hopkins Bloomberg School of Public Health and the International Center for Diarrheal Disease Research, Bangladesh (ICDDR,B). All participants gave signed informed consent prior to participation. The trial was registered at ClinicalTrials.gov (NCT00938600).

### Intervention

Vitamin D3 (cholecalciferol) 70,000 IU (1.75 mg) was administered directly by study personnel. The dose was selected to be intermediate between the doses previously studied in the only two rigorous single-dose vitamin D3 pharmacokinetic studies published at the time our study was designed (50,000 [10] and 100,000 IU [11]), thus providing reassurance in terms of probable safety as well as enabling coherent between-study comparisons. The vitamin D3 supplement (Vigantol Oil, Merck KGaA, Germany) was a liquid formulation (20,000 IU D3/mL). The batch of Vigantol Oil used in the study had a concentration of 20,697 IU/mL according to the manufacturer’s certificate of analysis (May, 2009). The stability of the vitamin D3 was established by independent testing of unused Vigantol Oil at the end of the study (June 2010) in the laboratory of Dr. Reinhold Vieth [12], which revealed a concentration of 19,300 IU/mL (96.5% of the labeled concentration). Participants were advised not to take other vitamin D-containing supplements during the study period, but were permitted to take other micronutrient supplements (including calcium). All pregnant participants were provided standard iron and folic acid supplementation.

### Follow-up

Study personnel assessed participants at least weekly. Non-pregnant participants who received only the single dose participated in weekly follow-up for 10 weeks; pregnant women in the single-dose group were assessed at least weekly until delivery, and then at least three times between delivery and discharge from the study at one-month post-partum. Visits involved a checklist of symptoms related to hypo- and hypercalcemia (decreased appetite, weight loss, vomiting, fever or chills, constipation, abdominal pain, excessive thirst, frequent urination, muscle weakness, back, arm, or leg pain, confusion, or depression), blood pressure measurement, and confirmation of fetal viability.

Abnormal urinalyses, hypertension, reported severe symptoms, or persistence of any mild symptomatic complaints (i.e., decreased appetite, weight loss, vomiting, fever or chills, constipation, abdominal pain, excessive thirst, frequent urination, muscle weakness, back, arm, or leg pain, confusion, or depression) for two consecutive visits prompted referral to the study physician for further evaluation. Participants were referred to an antenatal care physician at the maternity clinic for treatment of urinary tract infections, hypertension, or other medical problems that arose. Participants with obstetric complications were transported to a local tertiary-care hospital with advanced neonatal care facilities. All costs of medical and obstetric care were borne by the study.

### Specimen collection and biochemical analyses

**Figure 2:**
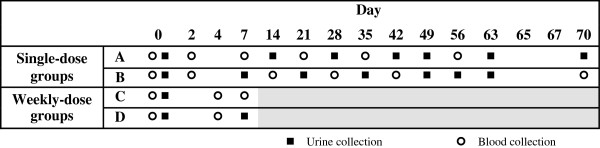
**Blood and urine specimen collection schedules. **Participants in “single-dose only” groups were randomized to one of two schedules (A or B) of specimen collection over a period 70 days. Participants in the “weekly dose” groups were similarly randomized to one of two schedules (C or D); however, the analysis of single-dose pharmacokinetics only included those specimens collected up to and including day 7, preceding administration of the 2nd vitamin D dose.

Participants provided up to six scheduled blood specimens and at least seven urine samples during the 10-week follow-up period beginning on the day of supplement administration (Figure 2). To limit the burden of specimen collection on each individual, yet still enable robust group-level pharmacokinetic and safety analyses, participants were assigned to one of two sampling schedules (A or B) to enhance coverage of the follow-up period (Figure 2). During the first week, specimens were collected at baseline and then additionally on either day 2 or 4 to monitor for possible early transient elevations in serum calcium and to minimize the chance of missing a possible early peak in [25(OH)D]. In the single-dose only groups, blood collection thereafter was scheduled predominantly in the first month because this was when the peak [25(OH)D] [11] and the highest risk of hypercalcemia were anticipated. In pregnant women in the single-dose only group, the specimen collection schedule was continued in the postpartum period if delivery occurred prior to 10 weeks from enrollment. In pregnant participants, venous cord blood samples were collected immediately following delivery of the placenta.

Serum samples (separated from maternal venous and umbilical vein blood) and random spot urine specimens were maintained at +4°C prior to same-day transfer to the laboratory. Sera were frozen at −20 °C. Aliquots for the 25(OH)D assay were shipped at ambient temperature from Dhaka to Toronto (25(OH)D is stable under a range of conditions). Total serum [25(OH)D] was measured with the Diasorin Liaison Total assay in the laboratory of Dr. Reinhold Vieth (Mount Sinai Hospital, Toronto) according to a method previously described [13]. This laboratory participates in and meets the performance targets of the International Vitamin D External Quality Assessment Scheme [14]. Mean within-run coefficient of variation (CV%) was 7.8% (5.8% for specimens with values < 150 nmol/L) and mean between-run CV% was 10.5% (9.0% for specimens <150 nmol/L). Ancillary serum and urine biochemical tests were performed using the AU640 Olympus Autoanalyzer (Olympus Corporation, Japan) at ICDDR,B.

The primary pharmacokinetic (PK) outcome measure was the serum [25(OH)D]; incremental changes from baseline (Δ[25(OH)D]) were calculated as an individual’s absolute [25(OH)D] at each visit minus her baseline [25(OH)D]. The primary safety-related outcome was maternal albumin-adjusted serum calcium concentration ([Ca]), calculated using a conventional formula: [Ca]+(0.02*(40-albumin)). The reference range for albumin-adjusted serum calcium was set at 2.10 – 2.60 mmol/L, the upper limit of which was a conservative threshold relative to those used by: the local laboratory in Dhaka (2.62 mmol/L), the US Institute of Medicine (IOM) 1997 dietary reference intakes (DRIs) for vitamin D (2.75 mmol/L) [15], and the IOM revised 2011 vitamin D DRIs (2.63 mmol/L) [1]. An albumin-adjusted serum calcium concentration >2.60 mmol/L prompted a repeat measurement on a new specimen as soon as possible. Confirmed hypercalcemia was *a priori* defined as albumin-adjusted serum calcium concentration > 2.60 mmol/L on both specimens, since hypercalcemia caused by vitamin D intoxication would not be expected to resolve within a few days without intervention.

The urinary calcium:creatinine ratio (ca:cr) was expressed as mmol Ca/mmol Cr, and 1.0 was considered the nominal upper limit of the reference range [16]. Any episode of urinary ca:cr>1.0 mmol/mmol prompted a repeat urine ca:cr measurement within one week. In addition, a ca:cr > 0.85 mmol/mmol that was also a 2-fold or greater increase over the lowest previously observed ratio in the same participant prompted repeat urine assessment. Persistent hypercalciuria was defined as ca:cr > 1.0 mmol/mmol on two consecutive tests, or on two non-consecutive measurements that occurred in the presence of persistent symptoms suggestive of possible hypercalcemia. Persistent hypercalciuria or persistent ca:cr > 0.85 mmol/mmol (under the conditions listed above) were indications for unscheduled measurement of serum calcium.

### Statistical analyses

Continuous outcome variables were described by means, standard deviations (SD), and 95% confidence intervals (95% CI). Non-normally distributed variables (including [25(OH)D]) were described by geometric means with 95% CI’s, medians and interquartile ranges (IQR), and were log-transformed for modeling. In the primary PK analysis, the following model-independent PK parameters were estimated for each individual in the single-dose only groups (N=31): 1) maximum observed [25(OH)D] (Cmax); 2) maximum observed Δ[25(OH)D] above baseline (ΔCmax); 3) timing of Cmax in days (Tmax); and 4) area under the Δ[25(OH)D]-time curve (AUC), which was interpreted as a global measure of vitamin D3 bioavailability. Individual participants’ AUCs were estimated manually by the trapezoidal method, and negative Δ[25(OH)D] values were zeroed so that the AUC represented the positive area above baseline. AUC was estimated for the first month to enable comparisons to other published PK studies [10,11]. AUC_28/35_ was calculated for either 0 to 28 days or 0 to 35 days, depending on the timing of the blood sampling (Figure 2); similarly, AUC_56/70_ was calculated for the period 0 to 56 days or 0 to 70 days. An individual’s average Δ[25(OH)D] during the first 28 days (ΔCavg_28_) was calculated by dividing AUC_28_ by 28; for between-study comparisons, this measure was expressed per 40,000 IU (1 mg) vitamin D3 by dividing ΔCavg_28_ by the dose administered (1.75 mg). Cmax, ΔCmax, Tmax, AUC_28/35_, and AUC_56/70_ were summarized within groups by geometric means and 95% CIs, and then log-transformed for one-way analyses of variance (ANOVA) to test for differences between the pregnant and non-pregnant groups. To plot the longitudinal change in [25(OH)D] over time using all available data (N=61), mean [25(OH)D] at each visit were predicted from a linear regression model using a random intercept for each participant, with each visit represented by its own fixed indicator variable. Cross-sectional differences in Δ[25(OH)D] between pregnant and non-pregnant groups at specific days of follow-up were compared by ANOVA. Changes in biochemical ([Ca] and Ca:Cr) and clinical outcomes from baseline were analyzed using generalized estimating equations (GEE) to account for repeated measures. The association between cord venous [25(OH)D] and the corresponding maternal [25(OH)D] closest in time to delivery was analyzed using Pearson correlation.

The target sample size of at least 12 analyzable participants per single-dose group was originally justified as follows: assuming two samples per subject (baseline and peak), a standard deviation for the ΔCmax of 20 nmol/L and an intra-subject correlation of 0.6, we anticipated that at least 12 women in each group would enable the estimation of the mean ΔCmax with 95% confidence bounds of ±10 nmol/L. In all analyses, *P* values less than 0.05 were considered to be statistically significant, with corrections for multiple comparisons using the Holm method, applied where appropriate [17]. Analyses were conducted using Stata version 10 and 11 (Stata Corporation, College Station, Texas).

## Results

In the single-dose only groups, follow-up for the full 10 weeks was completed in all non-pregnant (N=18) and pregnant (N=13) participants; however, the terminal serum sample for one non-pregnant participant (at day 56) was not suitable for analysis. Cord blood specimens were available for 12 of 13 pregnant participants in the single-dose only group. An additional 16 non-pregnant and 14 pregnant participants enrolled in weekly-dose groups contributed at least one [25(OH)D] value on or prior to day 7.

At baseline, pregnant participants had lower average [25(OH)D] than non-pregnant participants (Table 1); this was partly attributable to the design of the study, whereby some pregnant women were enrolled in the winter and all non-pregnant women were enrolled in the summer and fall (Table 2). Pregnant participants were generally younger, more likely to be married, and of a slightly lower socioeconomic status than non-pregnant participants (Table 2).

**Table 1:** **Changes in [25(OH)D] following a single dose of 70,000 IU vitamin D3 in non-pregnant and pregnant women in Dhaka, Bangladesh**^**a**^

	**All participants**	**Non-pregnant**	**Pregnant**	***P***^***b***^
**N** (all participants)	61	34	27	
**Baseline [25(OH)D] (N=61)**				
Mean [95% CI]	47 [42, 52]	54 [47, 62]	39 [34, 45]	0.010
Range	21, 96	27, 96	21, 95	
**Δ[25(OH)D], Mean [95% CI], nmol/L**				
Day 2 (N=27)	20 [15,25]	24 [17, 33]	15 [11,22]	0.037
Day 4 (N=27)	23 [18, 30]	24 [17, 34]	23 [16, 32]	0.800
Day 7 (N=29)	26 [21, 33]	25 [18, 34]	28 [20, 40]	0.134
Day 21 (N=14)	25 [18, 35]	21 [14, 33]	32 [20, 51]	0.003
Day 56 (N=12)	16 [11,23]	14 [9,21]	20 [12, 33]	0.101
**Participants in single-dose only groups**				
N (% followed more than 7 days)	31 (51%)	18 (53%)	13 (48%)	
**# Specimens per participant**				
Median	6	6	6	
Range	3, 6	3, 6	3, 6	
**Baseline [25(OH)D] (N=31)**				
Mean [95% CI]	48 [41, 56]	52 [42, 64]	43 [34, 55]	0.224
Range	21, 96	27, 96	21, 95	
**Tmax, days (N=31)**				
Mean [95% CI]	11 [7,18]	9 [4,17]	17 [10,29]	0.134
Range	2, 70	2, 70	2, 70	
**Cmax, nmol/L (N=31)**				
Mean [95% CI]	85 [77, 93]	87 [75, 101]	82 [72, 92]	0.500
Range	51, 164	51, 164	52, 116	
**ΔCmax, nmol/L (N=31)**				
Mean [95% CI]	30 [23, 39]	28 [18, 42]	33 [24, 46]	0.486
Range	2, 87	2, 87	9, 52	
**Area under the curve, nmol·d/L**^**c**^				
AUC_56/70_, Mean (N=30)	935	910	969	0.863
[95% CI]	[651, 1343]	[531, 1559]	[563, 1668]	
AUC_28/35_, Mean (N=31)	591	562	632	0.672
[95% CI]	[448, 780]	[383, 823]	[398, 1003]	
**ΔCavg**_**28/35 **_**per mg dose**^**d **^**(nmol/L/mg)**				
[95% CI]	12 [10,15]	12 [8,15]	14 [10,18]	0.370

^a. ^[25(OH)D] summary measures are geometric means with exponentiated 95% confidence intervals, unless otherwise indicated.

^b. ^One-way analysis of variance (ANOVA) test for difference between non-pregnant and pregnant groups.

^c. ^AUC was only estimated using data from participants with follow-up to the end of the interval of interest (i.e., 28/35 days or 56/70 days).

^d. ^Average [25(OH)D] over the first 28 or 35 days, per mg of the single vitamin D3 dose. Arithmetic means and 95% confidence intervals reported because these estimates had an approximately normal distribution.

**Table 2:** Personal and household characteristics of participants at enrollment

	**Single-dose only group**			**All participants**		
	**Non-pregnant**	**Pregnant**	***P***	**Non-pregnant**	**Pregnant**	***P***^***a***^
**# Enrolled**	18	13		34	27	
**Month of enrollment**						
July-August, 2009	18 (100 %)	5 (38 %)	<0.001	33 (97%)	5 (19%)	<0.001
Sept-Oct 2009	0	8 (62 %)		1 (3%)	8 (30%)	
February 2010	0	0		0	14 (52%)	
**Age (years)**, Mean (±SD)	23.9 (±3.8)	20.9 (±2.7)	0.022	24.2 (±4.1)	21.6 (±2.9)	0.006
**Married**	11 (61%)	13 (100%)	0.025	23 (68%)	27 (100%)	0.001
**Education level attained**						
None	1 (6%)	2 (15%)	0.750	3 (9%)	6 (22%)	0.293
Primary	11 (61%)	7 (54%)		21 (62%)	16 (59%)	
Secondary or higher	6 (33%)	4 (31%)		10 (29%)	5 (19%)	
**Husband’s education level**						
None	2 (18%)	3 (23%)	1.000	2 (9%)	4 (15%)	0.786
Primary	4 (36%)	4 (31%)		10 (43%)	13 (48%)	
Secondary or higher	5 (45%)	6 (46%)		11 (48%)	10 (37%)	
**Home ownership**	6 (33%)	1 (8%)	0.191	7 (21%)	2 (7%)	0.276
**House constructed from cement, brick or tile**^**b**^						
Floor	18 (100%)	11 (85%)	0.168	33 (98%)	22 (81%)	0.079
Walls	16 (89%)	10 (77%)	0.625	30 (88%)	18 (67%)	0.042
Roof	6 (33%)	6 (46%)	0.710	13 (38%)	7 (26%)	0.412
**Height (cm)**, mean (±SD)	149.7 (±3.7)	150.3 (±3.9)	0.685	150.8 (±4.3)	150.5 (±4.3)	0.758

^a. ^ANOVA for comparisons of continuous variables, Fisher’s exact test for categorical variables.

^b. ^In comparison to tin or natural materials (e.g., earth, bamboo).

### Pharmacokinetic outcomes

**Figure 3:**
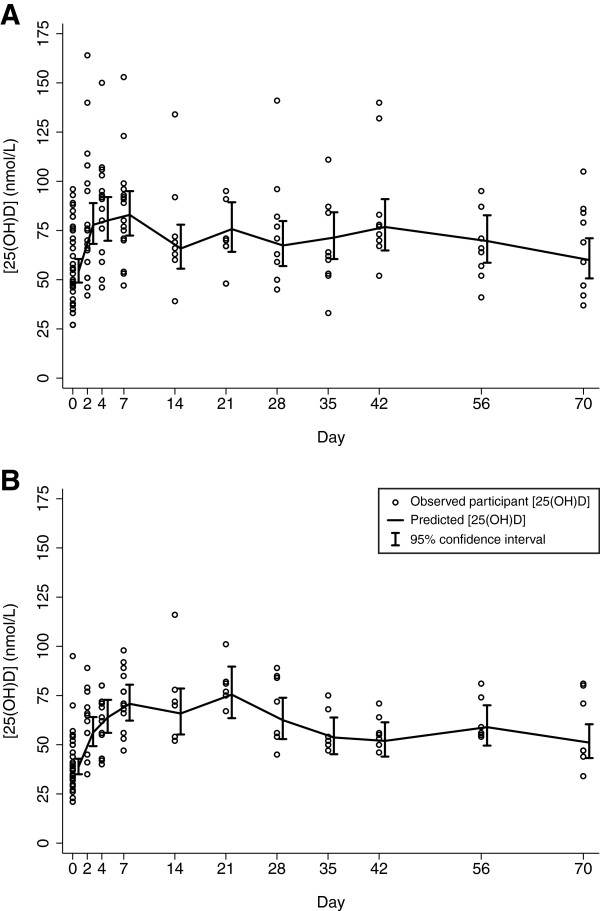
**Serum [25(OH)D] in non-pregnant (A) and pregnant (B) participants following administration of 70,000 IU vitamin D3 at day 0. **Predicted mean [25(OH)D] and 95% confidence intervals were estimated in a random-intercept regression model of ln[25(OH)D] as a function of time.

There was substantial inter-individual variation in the shape and magnitude of 25(OH)D responses to a single oral dose of 70,000 IU vitamin D3. However, the population-average pattern consisted of an abrupt increase in [25(OH)D] in the first week, followed by a peak within the first three weeks, and then a gradual return to baseline over the ensuing two months in both non-pregnant and pregnant participants (Figure 3). The average [25(OH)D] remained marginally above baseline at ten weeks after supplementation. There were minor differences between the pregnant and non-pregnant groups in the average Δ[25(OH)D] throughout follow-up (Table 1). In particular, [25(OH)D] rose more rapidly and the peak average occurred earlier in the non-pregnant group. This was demonstrated by the significantly greater Δ[25(OH)D] on day 2, the significantly lower Δ[25(OH)D] on day 21, and the slightly earlier occurrence of Tmax in non-pregnant vs. pregnant women (Table 1). Moreover, there was greater variance in the early Δ[25(OH)D] in non-pregnant vs. pregnant participants (Figure 3). The highest [25(OH)D] in any non-pregnant participant was 164 nmol/L, whereas the maximum in any pregnant participant was 116 nmol/L. On average, pregnant women had slightly lower absolute Cmax, but the mean maximal rise in [25(OH)D] (i.e., ΔCmax) and AUC were similar in pregnant and non-pregnant women (Table 1). Overall, the [25(OH)D] was an average of 19 nmol/L (95% CI, 14 to 25) higher than baseline during the first month after supplementation, which corresponded to a gain of approximately 12 nmol/L per mg of the vitamin D3 dose (Table 1).

### Safety outcomes

The supplement was tasteless and well tolerated and there were no supplement-related adverse events (Table 3). The stillbirth and newborn deaths were explained by medical problems, and there was no evidence that either was related to the vitamin D supplementation, given their timing (i.e., did not occur at peak [25(OH)D]) and the absence of biochemical evidence of vitamin D toxicity in the mother (Table 3). Postmortem examinations were not feasible in the study setting. Two other AEs resolved without complications and occurred in the absence of evidence of vitamin D toxicity (Table 3). Pregnancy and birth outcome metrics were consistent with expectations for the source population (Table 4).

**Table 3:** **Individual cases of elevated serum calcium or clinical adverse events among pregnant participants who received 70,000 IU vitamin D3**^**a**^

	**Event**	**Biochemistry**^**b**^					**Action**	**Outcome**
**ID**	**Description**	**Time of onset**	**Timing**	**Albumin –adjusted serum [Ca] (mmol/L)**	**Urine Ca:Cr (mmol/ mmol)**	**[25(OH)D] (nmol/L)**		
1	**Neonatal**: Neonatal death, secondary to respiratory failure, and pulmonary hemorrhage.	Delivery at 36 weeks gestation; day 42 of follow-up.	*Baseline*	2.43	0.05	95	Admitted to a neonatal intensive care unit.	Newborn died in hospital on postnatal day 3. No maternal complications.
			*Event*	2.38	0.74	71		
			*Post-event*	2.46	0.16	80		
			*Range*	2.38 to 2.48	0.05 to 0.74	71 to 116		
			*Cord blood*	2.69	–	72		
2	**Pregnancy**: Referral to hospital due to severe headache and vomiting.	32 weeks gestation; day 17 of follow-up.	*Baseline*	2.25	0.22	46	Admitted to tertiary-care hospital.	Discharged on day 2 of admission; no further complications. Delivered term infant; no complications.
			*Event*	2.26	0.74	85		
			*Post-event*	2.29	1.04	75		
			*Range*	2.24 to 2.37	0.22 to 1.04	46 to 85		
			*Cord blood*	2.51	–	49		
3	**Pregnancy**: Physician suspected irregular fetal heart rate; asymptomatic.	34 weeks gestation; day 28 of follow-up.	*Baseline*	2.30	0.39	54	Assessment at tertiary-care hospital.	Normal biophysical profile; normal pregnancy. No intervention required.
			*Event*	2.45	0.18	54		
			*Post-event*	2.50	1.48; repeat was 0.66	53		
	**Pregnancy**: Isolated albumin-adjusted serum [Ca] of 2.61 mmol/L.	1 week post-partum; day 70 of follow-up	*Event*	2.61	0.6	47	Repeat biochemistry was normal. No intervention required	Asymptomatic. No maternal or newborn complications (delivery occurred at 39 weeks gestation).
			*Post-event*	2.52	–	30		
			*Range*	2.3 to 2.61	0.14 to 1.74	47 to 70		
			*Cord blood*	2.61	–	30		
4	**Pregnancy**: Intrauterine fetal death, associated with placental abruption, hypertension and possible abdominal trauma.	35 weeks, 5 days gestational age.	*Baseline*	2.38	0.02	21	Fetus delivered by cesarean section.	Intrauterine fetal death, secondary to placental abruption. No further maternal complications
			*Event*	2.38	0.05	45		
			*Post-event*	2.46	0.05	46		
			*Range*	2.31 to 2.46	0.02 to 0.57	21 to 52		
			*Cord blood*	2.28	–	29		

^a. ^Table includes participants with at least one serum albumin-adjusted calcium concentration measurement greater than 2.60 mmol/L or a serious clinical adverse event at any time during follow-up, among participants in the single-dose only group.

^b. ^All values refer to serum concentrations in maternal venous blood except for the row labeled “cord blood”. “Range” refers to the participant’s range of values during the entire follow-up period.

**Table 4:** Pregnancy and newborn outcomes for pregnant participants who received only a single dose of 70,000 IU vitamin D at enrollment and were followed up to delivery

**N**	**13**
**Gestational age at birth**, weeks (by LMP) ^a^ Mean (±SD)	38.8 (±1.8)
Range	35.7 – 42.0
**Preterm, **n (%)	2 (15%)
**Birth weight**^**b **^(g)	
Mean (±SD) ^c^	2441 (±354)
Range (g)	1890 – 3005
n/N (%) Low Birth Weight	6/12 (50%)
**Delivery mode**, n/N (%) Cesarean section ^d^	8/13 (62%)
**Sex**, n (%) female	5 (38%)
**Live births**^**e**^	12/13
**Alive at 1 month of age**^**f**^	11/13

^a. ^In a sample of 113 deliveries at the study site (October 2009 to January 2010) for which there was a recalled first day of last menstrual period, the mean gestational age at birth was estimated to be 39.7 weeks (±2.2).

^b. ^Only includes the 12 liveborn infants.

^c. ^In a consecutive sample of 362 liveborn infants delivered at the study site (October 2009 to January 2010), the mean birth weight was 2780 g (±440).

^d. ^In a consecutive sample of 369 deliveries at the study site (October 2009 to January 2010), there were 199 cesarean deliveries (54%).

^e. ^There was one stillbirth. In a consecutive sample of 369 deliveries at the study site (October 2009 to January 2010), there were 7 stillbirths (2%).

^f. ^There was one neonatal death at 3 days of age.

**Figure 4:**
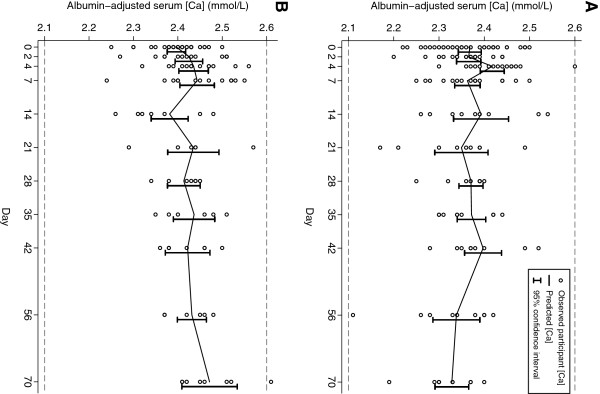
**Albumin-adjusted serum calcium concentration ([Ca]) in non-pregnant (A) and pregnant participants (B) following administration of vitamin D3 70,000 IU at day 0. **Dashed horizontal lines represent upper and lower bounds of the reference range. Predicted means and 95% confidence intervals were estimated in a linear regression model using GEE.

**Figure 5:**
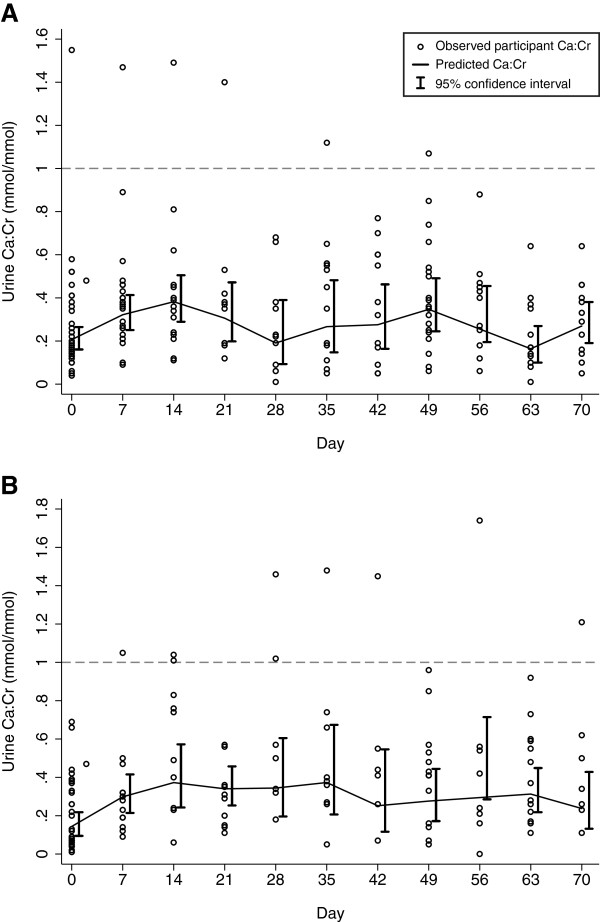
**Calcium:creatinine ratios (Ca:Cr) in spot urine specimens from non-pregnant (A) and pregnant participants (B) following administration of vitamin D3 70,000 IU at day 0. **Predicted means and 95% confidence intervals were estimated in a linear regression model using GEE, in which log-transformed Ca:Cr was modeled as a function of time.

Changes in average serum calcium concentrations (Figure 4) and urinary calcium excretion (Figure 5) occurred during the early phase of [25(OH)D] escalation. In non-pregnant participants, a transient increase in albumin-adjusted serum [Ca] from baseline was notable on day 4 (Table 5; Figure 4). The corresponding change in unadjusted total serum [Ca] was smaller and non-significant, and the raised adjusted [Ca] coincided with a lower average serum albumin on day 4 (difference versus baseline, -1.23 g/L; 95% CI, -2.12 to −0.34). In pregnant participants, there was an initial increase in albumin-adjusted [Ca] beginning on day 2 that persisted until nearly the end of the observation period (Figure 4), but the difference from baseline was only statistically significant on day 7 (Table 6). The unadjusted total [Ca] did not vary greatly from baseline and serum albumin remained relatively stable until the end of the 70-day follow-up, when many of the participants were post-partum.

**Table 5:** Serum calcium and urinary calcium:creatinine following single-dose vitamin D3 (70,000 IU) in non-pregnant participants

		**Total serum calcium conc. (mmol/L)**	**Albumin-adjusted serum calcium concentration (mmol/L)**			**Urine calcium:creatinine ratio (mmol/mmol)**				
**Follow-up time**	**N**^**a**^	**Mean (SD)**	**Mean (SD)**	**Range**	**# > 2.60**	**N**^a^	**Mean**^**b**^	**Median (IQR)**	**Range**	**# > 1.0**
0 days	34	2.45 (0.08)	2.37 (0.08)	2.22 to 2.5	0	34	0.21	0.19 (0.22)	0.04 to 1.55	1
2 days	18	2.44 (0.07)	2.36 (0.06)	2.2 to 2.44	0	–	–	–	–	–
4 days	16	2.48 (0.06)	2.42 (0.07) ^c^	2.3 to 2.6	0	–	–	–	–	–
2nd to 4th week *(days 7 to 27)*	34	2.44 (0.09)	2.37 (0.08)	2.17 to 2.54	0	47	0.34 ^d^	0.36 (0.23)	0.09 to 1.49	3
5th to 8th week *(days 28 to 55)*	27	2.44 (0.08)	2.38 (0.06)	2.25 to 2.52	0	53	0.28	0.35 (0.37)	0.01 to 1.12	2
9th to 11th week *(days 56 to 76)*	18	2.38 (0.09) ^e^	2.33 (0.08)	2.11 to 2.42	0	37	0.24	0.25 (0.26)	0.01 to 0.88	0
Total	147	2.44 (0.09)	2.37 (0.08)	2.11 to 2.6	0	172	0.26	0.3 (0.3)	0.01 to 1.55	6

^a. ^Number of specimens (there may have been multiple specimens for a single participant during a given follow-up period).

^b. ^Geometric means.

^c. ^Mean at day 4 was higher than baseline (+0.05; 95% CI, 0.03 to 0.07) and this remained statistically significant after correction for multiples testing.

^d. ^Means at days 7 and 14 were significantly higher than baseline, after correction for multiple testing.

^e. ^Mean at day 70 was significantly lower than baseline, after correction for multiple testing.

**Table 6:** Serum calcium and urinary calcium:creatinine following single-dose vitamin D3 (70,000 IU) in pregnant participants

		**Total serum calcium conc. (mmol/L)**	**Albumin-adjusted serum calcium concentration (mmol/L)**			**Urine calcium:creatinine ratio (mmol/mmol)**				
**Follow-up time**	**N**^**a**^	**Mean (SD)**	**Mean (SD)**	**Range**	**# > 2.60**	**N**^a^	**Mean**^**b**^	**Median (IQR)**	**Range**	**# > 1.0**
0 days	27	2.29 (0.08)	2.40 (0.05)	2.25 to 2.5	0	27	0.14	0.20 (0.32)	0.01 to 0.69	0
2 days	13	2.31 (0.09)	2.43 (0.07)	2.27 to 2.51	0	–	–	–	–	–
4 days	13	2.30 (0.08)	2.43 (0.06)	2.32 to 2.56	0	–	–	–	–	–
2nd to 4th week *(days 7 to 27)*	27	2.30 (0.10)	2.43 (0.09) ^c^	2.24 to 2.57	0	33	0.33 ^d^	0.31 (0.31)	0.06 to 1.05	3
5th to 8th week *(days 28 to 55)*	20	2.29 (0.07)	2.43 (0.05)	2.34 to 2.51	0	35	0.31^d^	0.41 (0.31)	0.05 to 1.48	4
9th to 11th week *(days 56 to 76)*	14	2.39 (0.11)	2.46 (0.06)	2.37 to 2.61	1	28	0.33 ^d^	0.36 (0.36)	0 to 1.74	2
Total	114	2.31 (0.09)	2.42 (0.07)	2.24 to 2.61	1	123	0.24	0.33 (0.36)	0 to 1.74	9
	**N**	**Mean (SD)**	**Mean (SD)**	**Range**	**# > 3.00**					
Cord Blood	12	2.57 (0.23)	2.68 (0.16)	2.28 to 2.9	0	–	–	–	–	–

^a. ^Number of specimens (there may have been multiple specimens from a single participant during a given follow-up period).

^b. ^Geometric means.

^c. ^Mean at day 7 was higher than baseline (+0.05; 95% CI, 0.01 to 0.08), which remained statistically significant after correcting for multiple testing.

^d. ^Means at days 7, 14, 21, 28, 35, 56, and 63 were significantly higher than baseline, after correcting for multiple testing.

**Figure 6:**
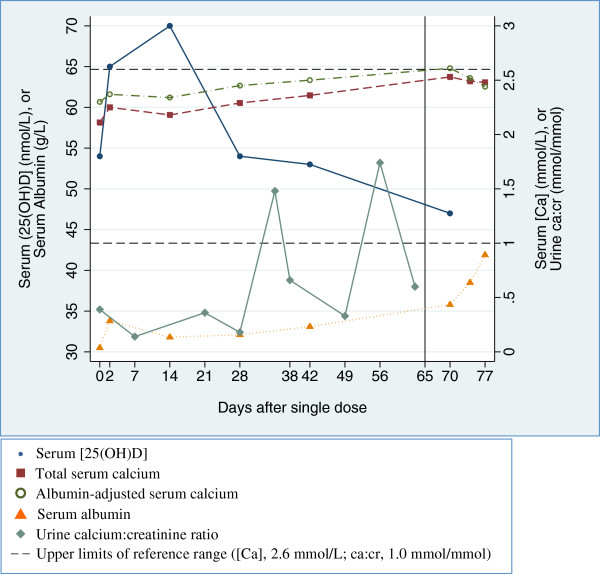
**Serum and urine biochemistry in a pregnant participant with two episodes of urine ca:cr > 1.0 mmol/mmol and one episode of serum albumin-adjusted [Ca] > 2.60 mmol/L. **Vertical line indicates timing of delivery at 39 weeks gestation.

There were no episodes of confirmed hypercalcemia according to the study definition, and no isolated albumin-adjusted [Ca] values greater than the recent IOM upper limit of normal of 2.63 mmol/L. One pregnant participant had a single albumin-adjusted [Ca] = 2.61 mmol/L at one-week postpartum (70 days after dose administration) corresponding to a normal total [Ca] (2.51 mmol/L; serum albumin concentration was 35.8 g/L) that was within the reference range on repeat testing 4 days later (Table 3; Figure 6). A further follow-up one week following the first abnormal result was also normal (albumin-adjusted serum [Ca] of 2.44 mmol/L). This participant also had two non-consecutive episodes of urinary ca:cr higher than 1.0 mmol/mmol during follow-up (Figure 6). Her serum biochemical patterns were consistent with the expected changes in the perinatal period, including a gradual increase in albumin-adjusted serum [Ca] towards the end of the antenatal period and a rapid increase in serum albumin in the post-partum period [18]. Furthermore, there was no temporal association between the rise in [25(OH)D] and either the occurrence of isolated peaks in urine ca:cr or the isolated elevated [Ca] (Figure 6).

None of the participants manifested persistent hypercalciuria according to the study definition, or using a more conservative threshold of 0.85 mmol/mmol. In non-pregnant participants, the Ca:Cr increased from baseline but differences were only statistically significant at day 7 and 14 (Table 5). In pregnant participants, the increases in average Ca:Cr above baseline were more persistent and were statistically significant on all days except day 42, 49, and 70 (Table 6). There was no overall difference in the average ca:cr between non-pregnant and pregnant participants (*P*= 0.857).

### Cord blood biochemistry

Among participants who had received a single dose at baseline and for whom cord blood specimens were collected (N=12), the geometric mean cord serum [25(OH)D] was 50 nmol/L (95% CI, 40 to 62; range, 29 to 80). All cord serum albumin-adjusted [Ca] were within the normal range. The cord ln[25(OH)D] was moderately correlated with the maternal ln[25(OH)D] closest to the time of delivery (Pearson rho=0.64, *P*=0.02), and the average ratio of cord:maternal [25(OH)D] (N=12) was 0.88 (95% CI, 0.76 –1.02).

## Discussion

This single-dose oral vitamin D3 pharmacokinetic study generated novel observations regarding the biochemical response to vitamin D3 in women of reproductive age in South Asia. Overall, we found that the average response was similar to that reported for non-pregnant adults in other geographic settings. The occurrence of the maximal mean [25(OH)D] in the first month was consistent with previous studies of single-dose vitamin D3 (1.25 to 15 mg) administered to non-pregnant adults in North America, Europe and Australia [10,11,19-23]. When expressed as a function of vitamin D3 dose (assuming the rise is linearly proportional to dose), the mean overall ΔCmax of 30 nmol/L (28 nmol/L in non-pregnant and 33 nmol/L in pregnant participants) represented an average maximal rise in [25(OH)D] of ~17 nmol/L per mg D3. This estimate was similar to those of previous studies from which relevant inferences could be drawn, in which the average ΔCmax ranged from 12 to 16 nmol/L per mg of vitamin D3 [10,11,19,20].

We are not aware of previous single-dose vitamin D3 pharmacokinetic studies in pregnancy to which the present findings can be directly compared. However, there are emerging data regarding the efficacy and safety of high-dose continuous regimens in pregnancy; for example, Hollis et al. reported that 4000 IU/day vitamin D3 initiated in the 2nd trimester yielded an increase in mean [25(OH)D] from 58 nmol/L to 111 nmol/L at delivery among women in South Carolina, without inducing hypercalcemia or other observed adverse effects [24]. In comparison, Vieth observed in non-pregnant adults that 4000 IU/day led to an increase in mean [25(OH)D] from 38 to 96 nmol/L at steady-state[16]. Thus, from a pharmacokinetic standpoint, the Hollis et al. findings are in accord with our conclusion that pregnancy does not substantially alter the 25(OH)D response to vitamin D3.

There was substantial inter-individual variability in 25(OH)D responses. Many participants demonstrated a rapid rise in [25(OH)D] during the first week, which is similar to the response to an acute dose of ultraviolet radiation exposure [25]; but distinct from the more gradual effects of other forms of exogenous vitamin D intake (e.g., oral D2 ingestion [10]). Several non-pregnant participants demonstrated peak [25(OH)D] as early as two days after supplement delivery, and there was notably wider variability in responses in the group of non-pregnant participants during the early escalation phase compared to pregnant participants. It is possible that the greater apparent variability was an artifact due to lower precision of the 25(OH)D assay at higher [25(OH)D], given the higher average [25(OH)D] in non-pregnant women. Higher concentrations of vitamin D-binding protein during pregnancy [[26] may have efficiently buffered the absorbed vitamin D3 and slowed its transport to the liver where it undergoes 25-hydroxylation [27]].

Vitamin D3 bioavailability (measured by mean AUC and dose-adjusted ΔCavg_28_) differed minimally between the non-pregnant and pregnant groups, and between-group differences were overshadowed by between-subject variability. The overall ΔCavg_28_ (i.e., estimated average [25(OH)D] rise from baseline in the first month, expressed per milligram of vitamin D3) was 12 nmol/L/mg based on an aggregate analysis of individual empiric AUCs. This result was the same as the ΔCavg_28_ of ~12 nmol/L/mg found in studies of non-pregnant adults using 50,000 IU and 100,000 IU [11]], and similar to an extrapolated estimate of 13 nmol/L/mg based on data reported for a single dose of 300,000 IU in elderly adults [19]. The ΔCavg_28_ provides a useful summary measure for between-study comparisons because most of a single ingested vitamin D3 dose is converted to 25(OH)D within one month [11]. The consistency of the present findings with ΔCavg_28_ estimates from previous studies supports the contention by Heaney et al. that 25(OH)D bioavailability is proportional to vitamin D3 input across a wide dose range (1.25 to 7.5 mg) [11]. Notably, ΔCavg_28_ extracted from a study by Cipriani et al. was somewhat lower (~ 8 nmol/L/mg) [20]. We speculate that the massive dose administered in that study (600,000 IU) saturated the hepatic 25-hydroxylase system, resulting in the engagement of subsidiary vitamin D catabolic pathways which reduced the 25(OH)D yield.

The single vitamin D3 dose of 70,000 IU did not provoke hypercalcemia or hypercalciuria in non-pregnant or pregnant participants, and available data indicated that adverse perinatal events were neither temporally nor mechanistically linked to vitamin D supplementation. An isolated serum [Ca] value above the reference range in one pregnant participant occurred in the early post-partum period, when albumin-adjusted [Ca] typically peaks [18]. This was not due to vitamin D toxicity because her [25(OH)D] at the time was 47 nmol/L and the [Ca] rapidly and spontaneously normalized. However, it is important to acknowledge that there were significant increases in average [Ca] and urine ca:cr. Changes in serum [Ca] were not reportedly significant in studies by Ilahi [11], Armas [10], or Romagnoli [19], but Cipriani et al. demonstrated that the administration of a single dose of 600,000 IU to healthy young adults caused an increase in serum [Ca] at 3 days, coinciding with peak serum concentrations of both 25(OH)D and the active metabolite, 1,25-dihydroxyvitamin D (1,25(OH)2D) [20]. Therefore, upward deflections in the serum and urine biomarkers of calcium homeostasis signaled a need to be cautious about the transient effects of large sudden influxes of vitamin D, and the risk of dose-dependent toxicity.

There were several limitations of this study. First, although we were able to closely monitor the participants to gain preliminary PK and safety data in this population, the small sample size limited the precision of effect estimates and comparisons of non-pregnant and pregnant participants. Moreover, we did not have adequate power to adjust for differences in the baseline characteristics of the pregnant and non-pregnant groups, although we did not expect minor variations in age or socioeconomic status to influence biochemical responses. Second, the low number of scheduled blood specimens collected from each individual compromised the precision of the estimates of individual-level PK parameters. The number was limited by available funds and the expected acceptability of the procedure by participants based on pre-study consultation with local community members. Third, the fixed timing of specimen collection had the disadvantage of leaving gaps in the [25(OH)D]-time curve where no data were available. Fourth, the study lacked an unsupplemented control group. The analysis was challenged by the substantial inter-individual variability in responses to supplementation, which was expected based on previous reports [28]. Several participants had fluctuating [25(OH)D], without a single clear peak and decline, and some manifested seemingly paradoxical responses, with initial declines in [25(OH)D] after D3 ingestion. These erratic patterns could not easily be explained on the basis of known vitamin D pharmacokinetics, but were most likely attributable to small-sample artifacts, biological variability in the absorption and metabolism of vitamin D, and inherent imprecision in the laboratory assessment of [25(OH)D]. Nonetheless, the data yielded coherent population-averaged interpretations that were consistent with published data from non-pregnant adults in other settings.

## Conclusions

Comparisons of pregnant (third-trimester) to non-pregnant participants, as well as comparisons to previously published PK studies in non-pregnant adults, suggested that the effects of pregnancy on the 25(OH)D response to vitamin D3 were relatively minor and did not substantially impact overall bioavailability. Likewise, we did not document any notable pregnancy-related hypersensitivity to a vitamin D dose of 70,000 IU in terms of its effects on calcium homeostasis. However, the unpredictability of the 25(OH)D response at the individual level, previous reports of adverse effects of large single doses [23], and the theoretical disadvantages of excessive fluctuations in vitamin D status [29] suggest that the use of large single or infrequent intermittent doses of vitamin D3 may be physiologically disadvantageous despite its practical appeal. Therefore, these data principally support the further investigation of single doses equal to or less than 70,000 IU in the context of intermittent (e.g., weekly or biweekly) antenatal dosing regimens.

## Abbreviations

PK: Pharmacokinetics; [25(OH)D]: 25-hydroxyvitamin D concentration; Δ[25(OH)D]: Change in [25(OH)D] from baseline; [Ca]: Albumin-adjusted serum calcium concentration; ca:cr: Urinary calcium:creatinine ratio; Cmax: Maximum observed [25(OH)D]; ΔCmax: Maximum observed Δ[25(OH)D] above baseline; Tmax: Timing of Cmax; ΔCavg_28_: Average Δ[25(OH)D] during the first 28 days after dose administration; AUC: Area under the Δ[25(OH)D]-time curve; AUC_28/35_: AUC calculated for 0 to 28 days or 0 to 35 days, depending on the timing of the blood sampling; AUC_56/70_: AUC for the period 0 to 56 days or 0 to 70 days; GEE: Generalized estimating equations.

## Competing interests

The authors declare that they have no competing interests.

## Authors' contributions

All authors were involved in the design of the study; DR and AM conducted the research; RR coordinated the laboratory analyses; DR performed the statistical analysis, wrote the manuscript, and had primary responsibility for the final content. All authors read and approved the final manuscript.
